# Feasibility of longitudinal *in vivo* monitoring of pulmonary disease progression in mouse models using laboratory-based x-ray dark-field CT

**DOI:** 10.1186/s41747-026-00789-w

**Published:** 2026-09-08

**Authors:** Jincheng Lu, Yizhou Bai, Li Zhang, Longchao Men, Peiyuan Guo, Wenjie Yu, Ruobing Liu, Xiaoting Xu, Peixuan Song, Haoyu Liu, Qianyue Ma, Runtao Deng, Zihao Zou, Yan Xu, Hongxia Yin, Zhenchang Wang, Zhentian Wang

**Affiliations:** 1 Department of Engineering Physics, Tsinghua University, Beijing, China; 2 Key Laboratory of Particle & Radiation Imaging (Tsinghua University) of Ministry of Education, Beijing, China; 3 Department of General Surgery, Beijing Tsinghua Changgung Hospital, School of Clinical Medicine, Tsinghua University, Beijing, China; 4 Department of Radiology, Beijing Friendship Hospital, Capital Medical University, Beijing, China

**Keywords:** Mice, Pulmonary alveoli, Pulmonary disease (chronic obstructive), Tomography (x-ray computed), x-rays

## Abstract

**Objective:**

Early alterations in pulmonary microstructure are central to the onset and progression of chronic obstructive pulmonary disease (COPD) and acute lung injury, yet these changes remain undetectable with conventional attenuation-based computed tomography (CT). Dark-field computed tomography is sensitive to small-angle x-ray scattering generated by intact alveolar structures; however, all prior *in vivo* studies have been cross-sectional and pseudo-longitudinal, precluding direct observation of disease evolution within the same subject. A longitudinal, microstructure-resolved imaging approach is needed to capture true disease trajectories, reduce inter-subject variability, and support preclinical therapeutic development.

**Materials and methods:**

We developed a dedicated dark-field CT imaging system and integrated workflow, including dose-optimized acquisition, a mouse-specific fixation device, motion compensation, pseudo-dark-field suppression, and deep learning–based three-dimensional lung segmentation, to support repeated imaging over 12 weeks in healthy, inflammatory-injury, and COPD mouse models.

**Results:**

The dark-field coefficient (µ_d_) showed distinct, model-specific temporal trajectories and appeared to reflect microstructural changes when attenuation (μ) remained stable. It exhibited pronounced left–right heterogeneity in the inflammatory-injury model and markedly different trajectories between two COPD mice under an identical protocol, underscoring inter-subject variability that the within-subject design captured. Overall, µ_d_ changed earlier than μ, and findings were consistent with terminal histology.

**Conclusion:**

Our results suggest the feasibility of long-term *in vivo* dark-field CT in preclinical lung studies. The dark-field coefficient shows promise as a potential noninvasive biomarker for early pulmonary damage, quantitative disease assessment, and therapy monitoring, supporting further investigation of longitudinal dark-field imaging in lung pathology and drug development.

**Key Points:**

- ***Question*** First laboratory-based longitudinal *in vivo* dark-field CT in mouse lung disease models over 12 weeks.
- ***Findings*** Dark-field signal showed more pronounced temporal variation than attenuation signal, with distinct model-specific trajectories.
- ***Relevance statement*** These findings establish a methodological foundation for longitudinal preclinical imaging of lung microstructure, which may support future translational research and the development of time-resolved imaging strategies for pulmonary disease.

**Graphical Abstract:**

**Figure:**
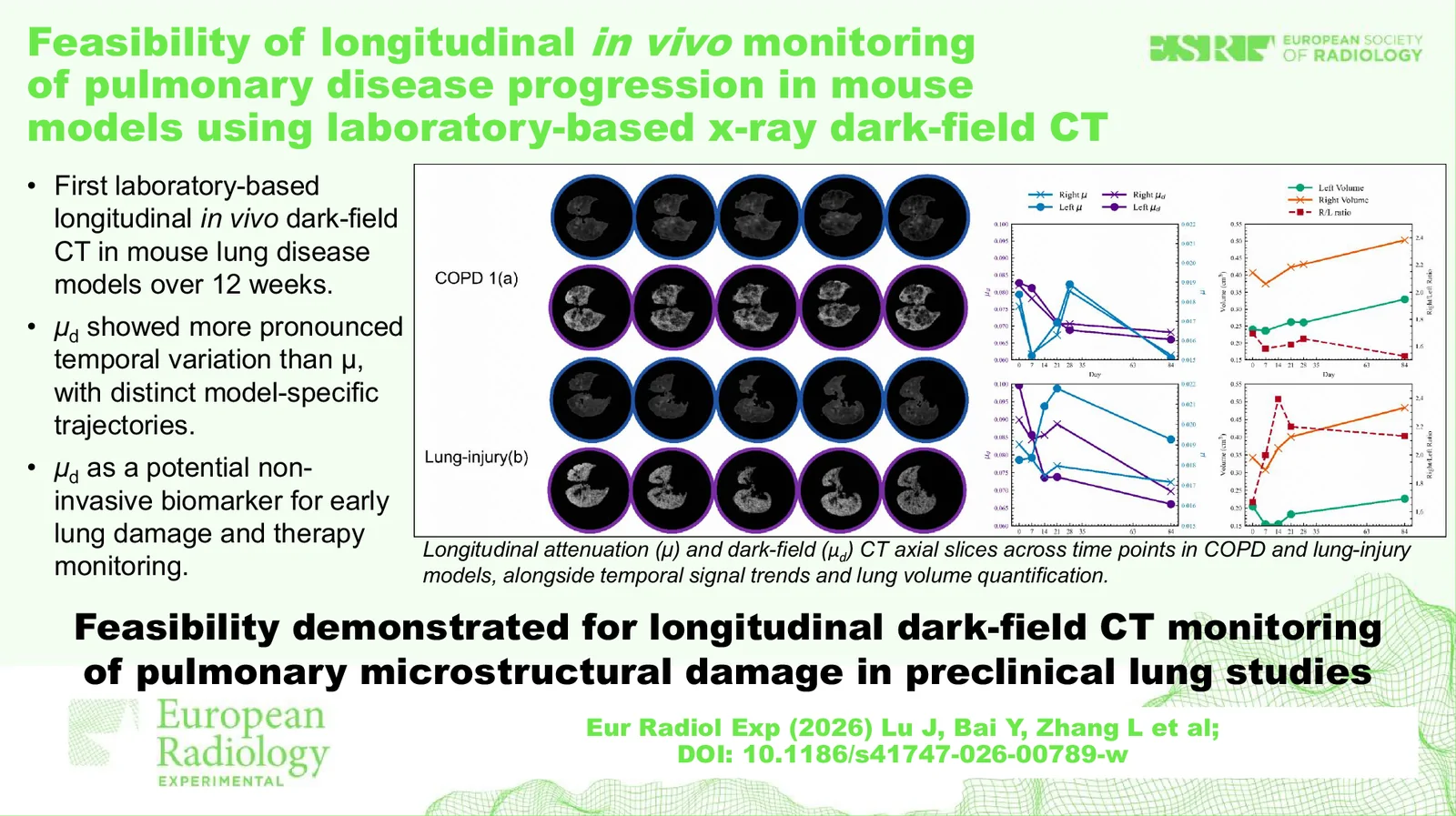

## Background

X-ray imaging reveals internal anatomy through differential attenuation [1], but the porous alveolar structure of lung tissue produces inherently weak attenuation contrast, limiting the sensitivity of conventional CT to early structural changes in diseases such as chronic obstructive pulmonary disease (COPD) [2]. Grating-based x-ray dark-field imaging captures small-angle scattering signals from microscopic structural heterogeneities [3, 4], offering high sensitivity to pathological alterations in alveolar microstructure [5–10]. As disease progresses and alveolar integrity is compromised, dark-field signals change pronouncedly even when attenuation remains largely unaffected [11–15], making dark-field CT a promising modality for early detection and monitoring of lung diseases [5, 16, 17].

Over the past decade, dark-field CT has advanced from preclinical disease models [5, 16–23] toward human-scale implementations [24–26] and respiration-resolved four-dimensional imaging [22]. However, the majority of existing studies remain cross-sectional, focusing on terminal stages or employing pseudo-longitudinal designs [21], which introduce inter-subject variability and hinder the tracking of intra-subject disease evolution. Prior studies have demonstrated *in vivo* dark-field CT of mouse lungs in various disease models, including emphysema [5, 7], lung cancer [9, 16], fibrosis [10], and radiation-induced injury [17, 23], but these investigations were limited to single time-point acquisitions. Guo et al [21] employed a pseudo-longitudinal design in which different animals were imaged at successive time points, but this approach inherently introduces inter-subject variability that obscures individual disease trajectories. More recently, How et al [22] reported respiration-resolved 4D dark-field CT, achieving temporal resolution within a single imaging session but not across extended follow-up periods. Longitudinal imaging of the same subject is essential for precisely characterizing microstructural changes over time while eliminating confounding inter-subject biological variability and demands dedicated imaging hardware and carefully optimized acquisition protocols. Beyond pulmonary applications, dark-field radiography has also been used to assess skeletal microstructure [27], indicating the broader applicability of small-angle scattering contrast.

This work establishes a complete experimental workflow that enables, for the first time, repeated dark-field CT imaging of the same mouse over an extended period in a laboratory setting. The integration of dose-optimized acquisition, motion compensation, pseudo-dark-field suppression, and deep learning–based segmentation into a robust longitudinal protocol—and its application to track within-subject disease evolution—represents the key advance over existing cross-sectional or pseudo-longitudinal studies. The workflow encompasses autocorrelation length optimization [28–31], joint longitudinal analysis of attenuation and dark-field signals, and a dual-contrast approach that monitors regional pulmonary microenvironmental changes, enabling non-destructive evaluation of disease progression and therapeutic response.

## Materials and methods

### Dedicated imaging system

All experiments were conducted using a dedicated dark-field CT system equipped with a Talbot–Lau grating interferometer developed at Tsinghua University, optimized for longitudinal *in vivo* mouse studies. The system was designed to maximize dark-field sensitivity at length scales relevant to murine alveolar microstructure, characterized by the scattering sensitivity S [32] (see Equation A1 in Supplementary Material Section A). The autocorrelation length was set to approximately 0.9 µm by adjusting the source-to-grating geometry and magnification. This value was chosen to match the characteristic dimensions of murine alveolar structures while avoiding dark-field signal saturation that arises at excessively large autocorrelation lengths (see Supplementary Material Section A for detailed optimization). The system simultaneously ensures adequate spatial resolution and a low radiation dose compatible with repeated imaging.

**Fig. 1:**
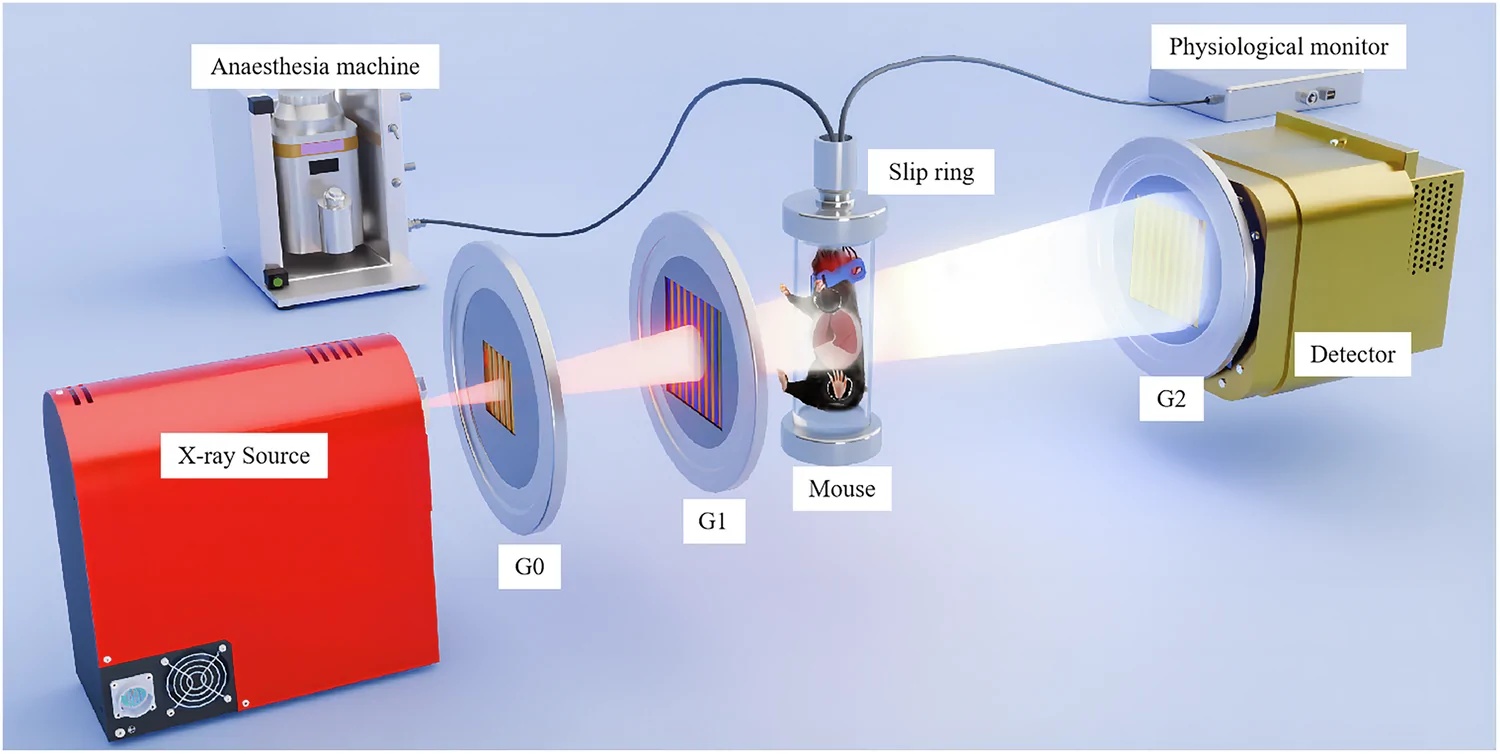
Imaging setup for longitudinal *in vivo* dark-field CT. Mice were anesthetized with inhaled isoflurane and physiologically monitored during CT. CT, Computed tomography

The setup comprises a Hamamatsu L12161-07 microfocus x-ray source (Hamamatsu Photonics K.K.), operated at 53 kVp and 500 µA, with a focal spot size of 20 µm. Image acquisition was performed using an energy-integrating detector (Photonic Science sCMOS 16MP 95), equipped with a 40 µm-thick Gadox scintillator and a pixel size of 16.4 µm. The Talbot–Lau grating interferometer consisted of three gratings fabricated on silicon substrates [33, 34], each with a period of 4.8 µm and a duty cycle of 0.5 [21]. The phase grating (G1) was designed to provide a π phase shift at the design energy of 30 keV. The distance between adjacent gratings was set to 0.139 m, corresponding to the first fractional Talbot order at this energy. A schematic of the experimental setup is presented in Fig. 1. During imaging, the mouse was immobilized on a CT rotation stage, which could be remotely translated in both horizontal and vertical directions. This allowed the mouse to be moved out of the field of view for the acquisition of reference images. More methodological details are provided in Supplementary Material Section A.

### Mouse preparation

We enrolled three mouse cohorts to characterize longitudinal signal changes across lung disease models: healthy (*n* = 1), inflammatory lung injury (*n* = 1), and COPD (*n* = 2). The two COPD mice were included to assess inter-subject variability in disease evolution under an identical induction protocol. To establish disease mouse models, 8-week-old male C57BL/6 mice (Spf Biotechnology Co., Ltd.) were used in this study. Following a 1-week acclimatization period, mice received an intratracheal instillation of 50 µL elastase solution (3 U per mouse; Acmec Biochemical Co., Ltd.) diluted in phosphate-buffered saline (PBS) to induce emphysematous lung injury [35] as well as intratracheal instillation of PBS alone, which served as a vehicle control. Post-mortem histological examination revealed that this mouse developed inflammatory lung injury, likely as a consequence of procedural irritation during intratracheal administration.

Dark-field CT scans were performed at baseline (day 0) and on days 7, 14, 21, 28, 35, 63, and 84 following elastase administration to monitor disease progression. Mice were anesthetized during imaging and returned to a controlled housing facility upon recovery. All animal procedures were conducted in strict accordance with the national guidelines for the ethical review of laboratory animal welfare (GB/T 35892-2018).

Mice were secured in custom-designed polystyrene fixation tubes with lateral openings for limb support, minimizing motion during upright imaging (see Supplementary Material Section A).

Vital signs were continuously monitored using a small-animal monitoring system (Starr Life Sciences Co.) supplemented by real-time camera observation. Inhalational isoflurane anesthesia was delivered *via* tubing integrated through a slip ring, enabling uninterrupted anesthesia during CT acquisition.

### Anesthesia parameters

All anesthesia experiments were performed at an ambient temperature of 23 °C. Following the mechanical stabilization of the animal, appropriate anesthesia parameters were optimized to ensure physiological stability and animal safety during prolonged sedation. Based on extensive empirical evaluation, we found that in healthy mice, continuous anesthesia with 1.5% isoflurane allows up to 7 h of safe imaging without mortality. However, in mice with pulmonary disease, the safe anesthesia window was reduced to approximately 4 h. Additionally, we observed that a respiratory rate below 10 breaths per minute was predictive of imminent mortality. During imaging, the respiratory rate of anesthetized mice was generally maintained between 20 and 40 breaths per minute. To minimize anesthesia-related complications, particularly those affecting the eyes due to prolonged airflow exposure, we adjusted the oxygen flow rate to 0.35 L/min and applied a thin layer of petroleum jelly to the ocular surface immediately after anesthesia induction.

### *In vivo* data acquisition

Each mouse was placed in the fixation tube and mounted vertically on the rotation stage. Acquisitions were performed at a magnification of approximately 1.3 (effective autocorrelation length: 0.9 µm; see Supplementary Material Section A for parameter optimization).

To accommodate the requirements of *in vivo* imaging—minimizing mechanical motion time and detector readout dead time—a three-step phase-stepping protocol was adopted [36]. To further mitigate ring and periodic artifacts, 200 projection angles were uniformly distributed according to the golden-angle scheme [37].

The detector operated in 2 × 2 binning mode (effective pixel size: 32.8 µm), with 5 s exposure per phase step, yielding a total scan time of approximately 60 min and an effective dose of approximately 540 mGy per scan, within acceptable limits for longitudinal imaging [38]. Further details on acquisition parameters and dose measurements are provided in Supplementary Material Section B.

For phase retrieval, a reference set of phase-stepping images (without the sample) was acquired every 100 projection angles. During these reference acquisitions, the mouse was translated out of the x-ray beam to remove it from the optical path.

### Tomographic reconstruction

Attenuation and dark-field signals were extracted from each phase-stepping dataset using Fourier analysis [39, 40]. Both signals can be reconstructed in the same manner by standard algorithms [4, 41]. To improve reconstruction accuracy, a pixel-wise pseudo-dark-field correction was applied to remove beam-hardening-induced artifacts [42]. Polymethyl methacrylate (PMMA) was selected as the calibration material for this correction because it is a homogeneous, amorphous polymer that does not produce small-angle x-ray scattering [43, 44]; consequently, any dark-field signal measured through PMMA is entirely attributable to beam-hardening artifacts, making it an ideal non-scattering reference for pseudo-dark-field calibration. To compensate for the gradual downward displacement of the lungs during upright imaging, a view-by-view motion correction based on Otsu thresholding [45] and displacement modeling was applied. Details on beam-hardening correction and motion correction are provided in Supplementary Material Section B.

**Fig. 2:**
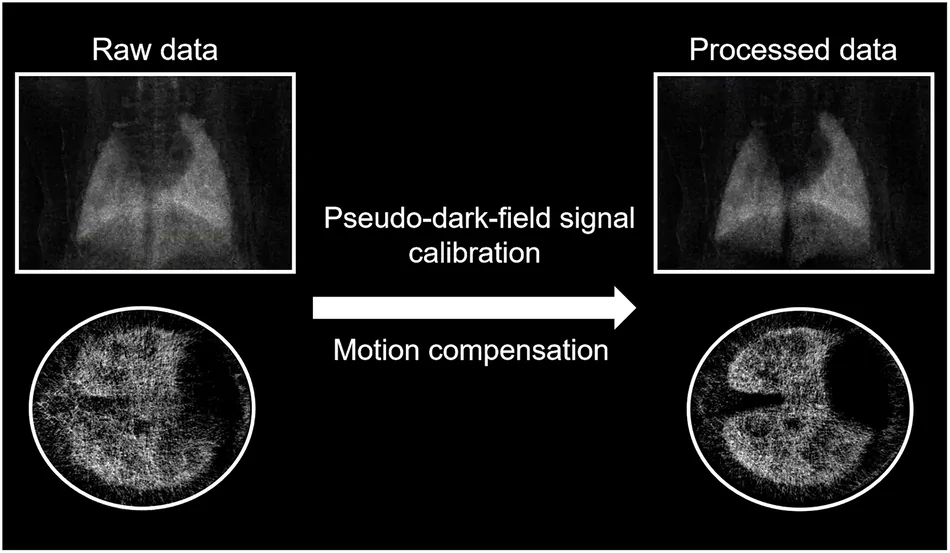
Schematic of the reconstruction workflow. The lower left panel shows the unprocessed reconstruction result, while the lower right panel shows the processed reconstruction result

To minimize reconstruction artifacts from insufficient angular sampling [46], we employed Simultaneous Iterative Reconstruction Technique (SIRT) *via* the TIGRE toolkit [47], with a volume of 1,000³ voxels (25 µm isotropic). Figure 2 validates the effectiveness of the proposed processing workflow.

### Postprocessing

To further reduce image noise while preserving structural edges, a cross-channel-based bilateral filter was applied to the reconstructed dark-field volumes [48], with a filter radius of 6 voxels, a spatial-domain standard deviation σ_s_ = 1, and an intensity-domain standard deviation *σ*_*i*_ = 0.1. To maximize data fidelity, all datasets from each scan were subjected to rigorous quality control; when pervasive stripe artifacts were present across the majority of projection views in a dataset, the entire CT reconstruction from that dataset was excluded from subsequent quantitative analyses. The detailed quality control procedure is described in Supplementary Material Section C.

For longitudinal comparison, a three-dimensional whole-lung analysis was adopted, as protease-induced COPD produces diffuse damage [49–51] and consistent slice matching across sessions is inherently difficult.

Automated lung segmentation was achieved by fine-tuning an nnU-Net model [52] on approximately 3,000 manually annotated slices, as existing networks did not generalize to our image characteristics (see Supplementary Material Section D for details).

All segmentations underwent slice-wise quality control with targeted manual corrections applied to approximately 10% of slices, primarily in regions where automated segmentation included extrapulmonary structures or missed peripheral lung tissue. Because all final segmentations were visually verified and corrected by an experienced operator, the quantitative results reflect human-validated masks. Analyses were stratified into left and right lung compartments. Accurate lung volume determination is critical for reliable quantitative dark-field analysis, as recently emphasized by comparisons of CNN-based, shape-based, CT-based, and pulmonary-function-based methods [53].

### Quantitative analysis

From the longitudinally segmented CT volumes, we extracted the time-series trajectories of attenuation (µ) and dark-field coefficient (µ_d_) at each imaging time point. Here, µ_d_ denotes the linear diffusion coefficient obtained by tomographic reconstruction of the dark-field projection signal -ln(*V*_*s*_/*V*_*r*_), where *V*_*s*_ and *V*_*r*_ are the sample and reference visibilities, respectively. This quantity is physically equivalent to the linear diffusion coefficient described by Taphorn et al [54] and quantifies the small-angle scattering power per unit path length. These signals were analyzed to characterize intra-subject changes over time.

For all measurements, we used the median rather than the mean for two reasons: (1) the lung masks inevitably include blood vessels and airways that contribute little or no dark-field signal, and (2) the dark-field channel is more susceptible to noise and outliers, for which the median provides a more robust estimate [55].

### Histology

At the conclusion of the final longitudinal imaging, mice were euthanized by overdose of isoflurane anesthesia. Following euthanasia, the lungs were perfused with 10% neutral-buffered formalin *via* tracheal instillation to ensure uniform fixation. The entire heart–lung block was then immersed in formalin to prevent flotation of the lung tissue. After 24 h of fixation, the samples were transferred to ethanol for dehydration, embedded in paraffin, and sectioned at a thickness of 10 µm. Histological slices were stained with hematoxylin and eosin and digitized using a high-resolution slide scanner for subsequent analysis.

## Results

### Projection-level longitudinal signal trends

**Fig. 3:**
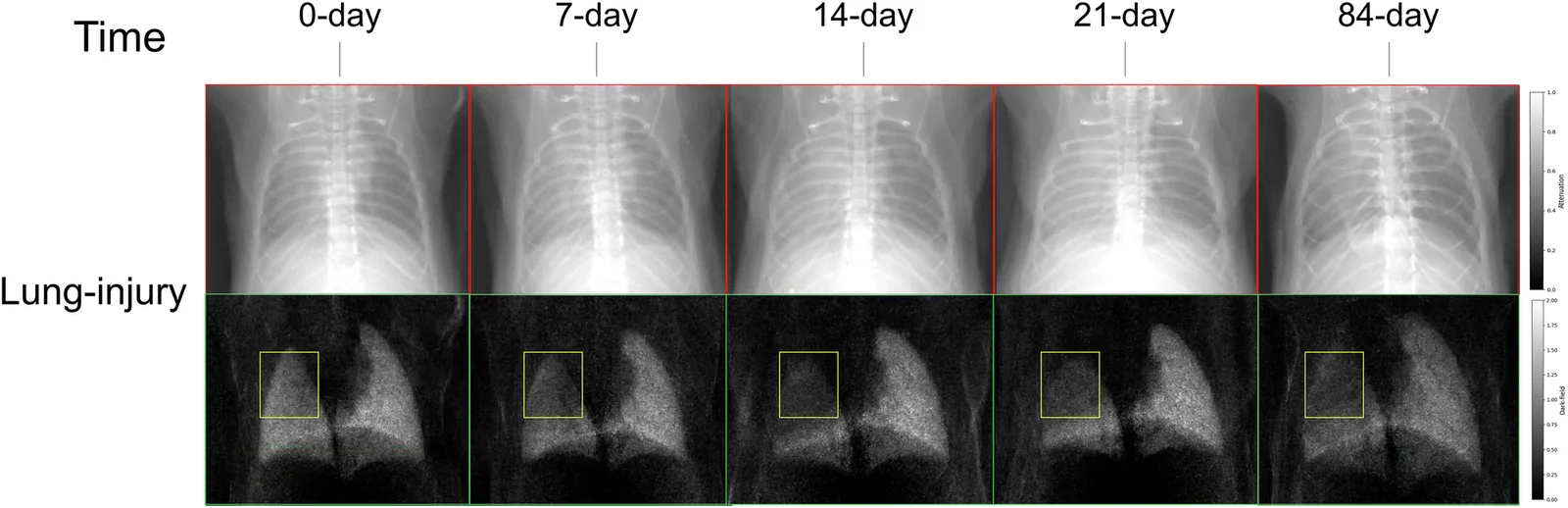
Representative longitudinal digital radiography images of a lung injury model

Figure 3 presents representative longitudinal study results from a mouse with inflammatory lung injury. For direct visual comparability, all images of the same modality were displayed using identical grayscale windows: attenuation µ in the range [0,1] mm^-1^ and dark-field µ_d_ in the range [0,2] mm^-1^. The projection viewing angle (animal orientation) was kept as consistent as possible across time points. In the yellow-marked region of the left lung, the dark-field signal decreased rapidly during days 0–21, while the right lung signal remained largely unchanged. Between days 21 and 84, both the red-marked region and the right lung exhibited a slight decline in dark-field signal, whereas no clear trend was observed in the attenuation images. Overall, the dark-field signal in this inflammatory lung injury mouse demonstrated pronounced temporal and spatial dependence in the projection images.

However, longitudinal assessment based on digital radiography is limited by its intrinsic characteristics and does not allow precise quantification; therefore, we further performed CT-level analyses.

### CT-level longitudinal signal trends

**Fig. 4:**
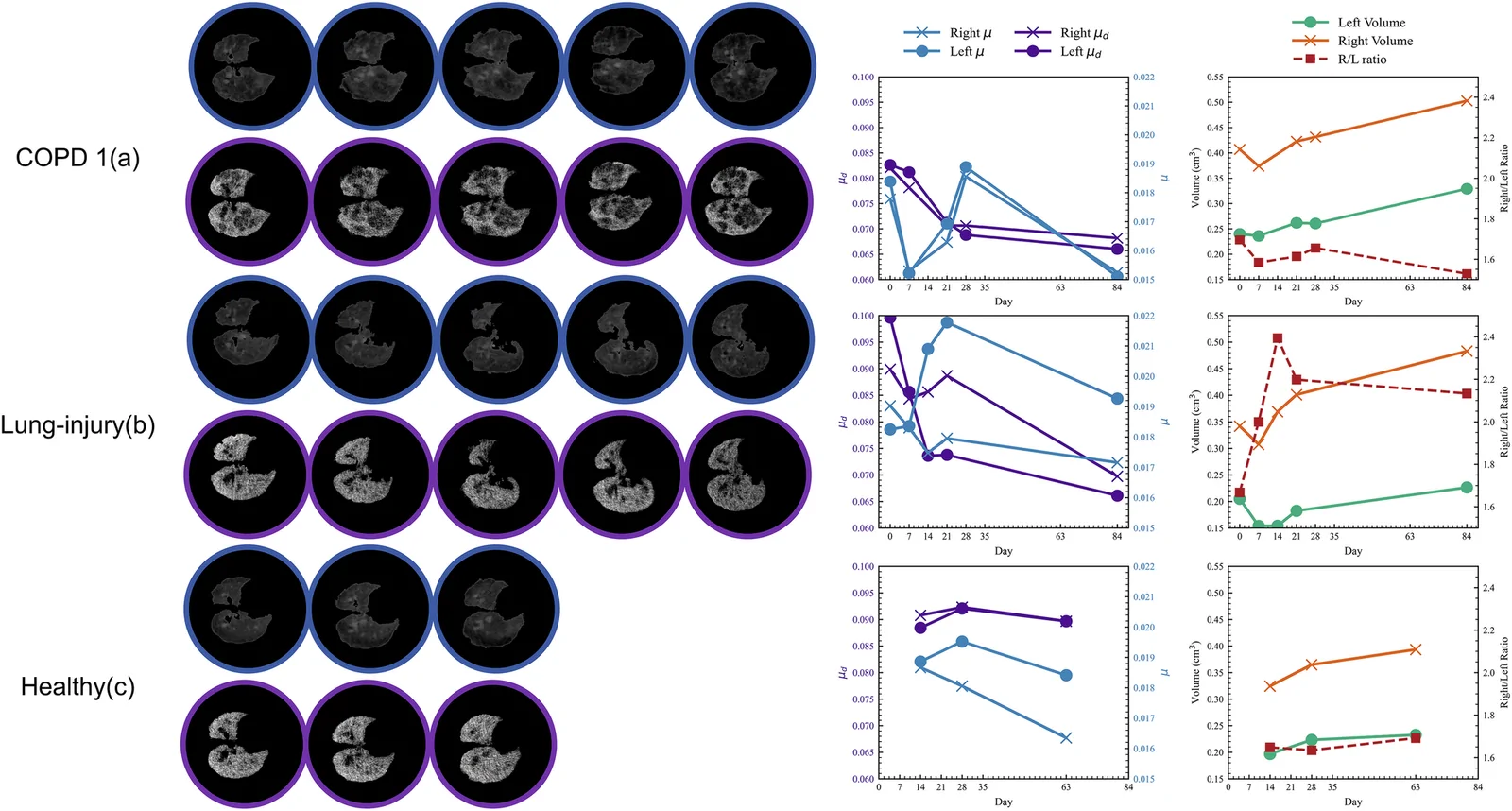
Shown on the left are longitudinal computed tomography axial slices of attenuation (µ) and dark-field (µ_d_) across imaging time points. **a**–**c** The COPD-1 mouse (**a**), the lung-injury mouse (**b**), and the healthy control mouse (**c**). COPD, Chronic obstructive pulmonary disease

Following the analysis methods described in the “Materials and methods” section, and the data quality control procedures described in Supplementary Material Section C, Fig. 4 presents longitudinal axial CT slices for each mouse. For each animal, slices were manually aligned to correspond as closely as possible to the same pulmonary level. Shown are (a) COPD-1 mouse, (b) lung-injury mouse, and (c) healthy mouse.

From Fig. 4a, qualitative inspection shows no clear temporal trend in the attenuation channel. By contrast, the dark-field channel exhibits a progressive increase in the area fraction of low-signal voids within the bright parenchymal signal, accompanied by an overall decline in dark-field intensity over time. From Fig. 4b, qualitative inspection indicates that the left-lung dark-field signal decreases initially and then recovers, whereas the right lung remains essentially stable, evidencing marked left–right heterogeneity. In Fig. 4c, neither the attenuation nor the dark-field channel exhibits a clear temporal trend.

We then performed a more quantitative analysis of the longitudinal CT data. The quantitative results are shown in the right panels of Fig. 4; the horizontal axis denotes the elapsed time (days) since the first imaging session. The left panel shows the median signal over time (blue: dark-field µ_d_; orange: attenuation µ), and the right panel shows the lung volume. All results are derived from the lung segmentation data. Quantitatively, all three models exhibit a common trend in lung volume—an initial decline followed by a subsequent increase over time across the available time points. However, the temporal trends of the median dark-field and attenuation signals differed markedly across models.

As shown in Fig. 4, we focused on left–right separated analyses, because whole-lung results largely mirror the combined behavior of the two lungs. Across all mice, right-lung volume exceeded left-lung volume, with a right/left volume ratio of approximately 2, although this ratio varied with disease model and time.

In the COPD-1 mouse (Fig. 4a), dark-field (µ_d_) and attenuation (µ) trends were largely concordant between the left and right lungs, and consistent with the overall whole-lung trajectory. In the early post-induction period, the observed volume loss may reflect focal atelectasis reducing ventilated parenchyma, while the decreases in both µ and µ_d_ could be attributed to overdistension of the remaining aerated regions. As the early phase resolved and growth resumed, lung volume increased substantially. This recovery was accompanied by a rise in µ, possibly due to residual inflammatory exudates, while the progressive decline in µ_d_ may reflect continued alveolar destruction. From day 28 to day 84, the continued volume increases with concomitant reductions in both signals are consistent with aging-related changes and COPD progression. Side-specific volume trends also allowed identification of the lung in which pathological changes were more pronounced.

In the lung-injury mouse (Fig. 4b), pronounced lateral heterogeneity was observed. Early after induction, the left lung showed a precipitous drop in µ_d_ and a rapid rise in µ, possibly reflecting acute inflammation, whereas the right lung showed gradual decreases in both channels, which may be related to focal atelectasis. Because right-lung volume was substantially larger, whole-lung attenuation statistics exhibited an apparent decrease followed by an increase. Between day 14 and day 21, both lungs indicated stabilization and partial recovery. Thereafter, with somatic growth, lung volume continued to increase while both µ and µ_d_ declined. The right-to-left volume ratio may suggest that the right lung disproportionately supported ventilation in the early phase due to severe left-lung involvement; after day 14, the decreasing ratio may suggest progressive recovery of the left lung.

In the healthy control mouse (Fig. 4c), both µ_d_ and µ showed small-amplitude oscillations and were broadly consistent between the two lungs, in line with normal growth. Lung volume increased gradually but remained lower than in the COPD mice.

### Inter-subject variability

**Fig. 5:**
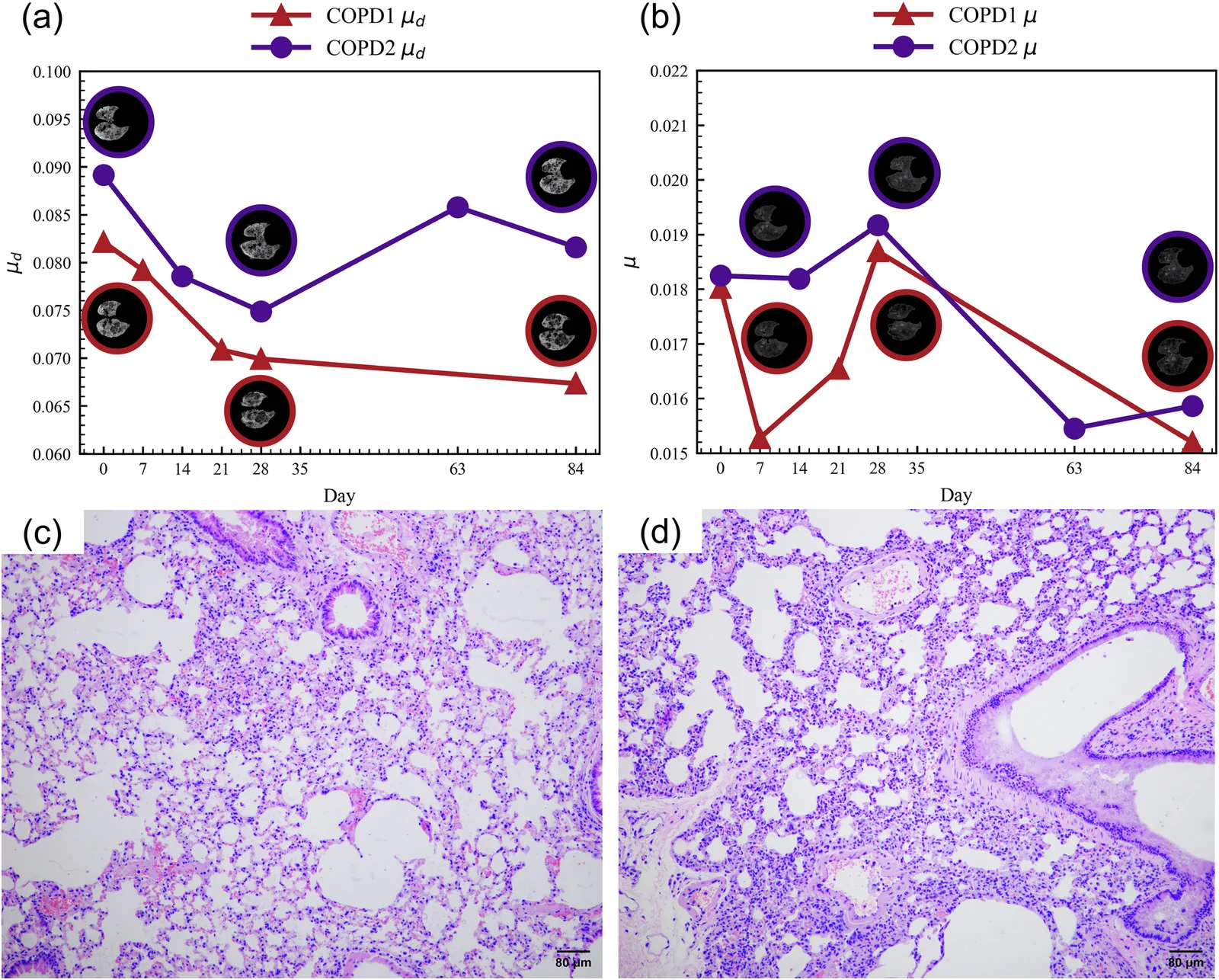
**a**, **b** The longitudinal temporal trends of dark-field (µ_d_) and attenuation (µ) signals for the COPD-1 and COPD-2 mice, respectively. **c**, **d** Hematoxylin and eosin-stained histological sections. COPD, Chronic obstructive pulmonary disease

Even among littermates subjected to the same induction protocol, intrinsic physiological differences yield appreciable within-group variability [56]. Therefore, we conducted a longitudinal study of two COPD model mice generated with the same protocol to characterize inter-subject variability. Results are shown in Fig. 5a (COPD-1 mouse) and 5b (COPD-2 mouse).

Because diffuse COPD exhibited left–right homogeneity, as shown in Fig. 4a, we focused on whole-lung signals in subsequent analyses. As illustrated in Fig. 5a, b, the whole-lung dark-field (µ_d_) and attenuation (µ) were therefore used to characterize disease evolution. By contrast, marked inter-subject differences were evident: in Fig. 5a, µ_d_ declined monotonically while µ followed a decrease–increase–decrease pattern; in Fig. 5b, µ_d_ exhibited a decrease–increase–decrease sequence, whereas µ showed an increase–decrease trend with a subsequent mild rebound. These findings indicate that, even under an identical induction protocol, inherent biological variability can lead to substantially different signal trajectories across subjects.

In COPD-2, disease evolution differed modestly from COPD-1. During the early phase, the increase in attenuation (µ) alongside a sustained decrease in dark-field (µ_d_) may reflect cellular exudation. Between day 28 and day 63, the decrease in µ and concomitant increase in µ_d_ could be consistent with progressive resolution of inflammation and gradual lung growth. In the final interval, the renewed decline in µ_d_ and slight rise in µ may suggest continued volume expansion together with possible fibrotic remodeling. Relative to COPD-1, the observations in Fig. 5d may suggest that COPD-2 was at a relatively earlier disease stage, potentially with a more fibrotic component; the smaller lung volumes in COPD-2 are consistent with this interpretation. Histology (Fig. 5c, d) showed greater inflammatory cell infiltration in COPD-2 than in COPD-1, which supports this observation.

## Discussion

This work presents a dedicated grating-based dark-field CT system for mouse lung imaging and reports the first laboratory-based longitudinal dark-field CT study in mice, accompanied by an automatically segmented dataset with trained weights to support three-dimensional quantitative analysis. The central advance lies in the longitudinal within-subject imaging design, which eliminates the inter-subject variability inherent in pseudo-longitudinal approaches and enables direct observation of disease evolution in individual animals. By leveraging the complementary contrast of dark-field (µ_d_) and attenuation (µ), the approach may enable detection of early parenchymal alterations and a finer-grained characterization of disease evolution. The longitudinal design mitigates the inter-subject variability that often confounds pseudo-longitudinal studies, providing a noninvasive imaging framework to evaluate time-resolved disease progression and therapeutic response.

Technically, this capability relies on the Talbot–Lau three-grating interferometer, which enables the extraction of a dark-field signal complementary to attenuation. However, several challenges remain.

The grating interferometer intrinsically reduces photon efficiency, particularly through the analyzer grating (G2), which blocks approximately half of the post-sample photons. Additionally, the laboratory prototype poses challenges for longitudinal reproducibility: identical acquisition conditions are difficult to guarantee across sessions, mechanical motion overhead prolongs scans, and upright positioning introduces alignment variability. These factors, combined with the limited sample size, preclude large-scale statistical analysis.

These limitations could be addressed by dedicated small-animal gantry systems [57, 58] and advanced acquisition strategies [59–61], which would improve reproducibility, photon efficiency, and throughput for biological and translational applications. We note that the pixel-wise pseudo-dark-field calibration assumes a universal relationship between attenuation and visibility reduction. Because the spectral beam-hardening characteristics of bone and soft tissue differ, slight residual pseudo-dark-field artifacts may remain in regions with complex bone–tissue interfaces. However, this residual effect is systematic and spatially consistent across time points and therefore does not compromise the validity of longitudinal trend analysis. However, this effect is systematic and spatially consistent across time points and therefore does not compromise the validity of longitudinal trend analysis.

The radiation dose of approximately 540 mGy per scan in this study is comparable to the 4D respiratory-gated micro-CT protocol evaluated by Berghen et al [38], who reported that weekly scanning at this dose level over 5 weeks produced subclinical hematologic changes (reductions in platelets and lymphocytes) but did not affect lung tissue morphology, disease progression, or imaging-derived biomarkers in murine models of metastasis, inflammation, and fibrosis. Although our study involved up to eight scans (cumulative dose ~4,320 mGy), the imaging intervals were more widely spaced (up to 12 weeks rather than 5 consecutive weeks), allowing greater recovery time between exposures. Nevertheless, radiation-induced confounding cannot be entirely excluded, particularly for inflammatory readouts. Future studies will incorporate radiation-exposed control groups and explore dose-reduction strategies, such as the ~200 mGy three-dimensional protocol described by Berghen et al [38], which eliminated all detectable biological effects while maintaining diagnostic image quality.

The inflammatory lung injury observed in the PBS-instilled mouse was identified retrospectively from terminal histology rather than from a pre-defined disease-induction protocol. The onset, severity, and nature of the inflammatory response were not independently validated by additional readouts such as bronchoalveolar lavage or inflammatory biomarkers, which limit the mechanistic interpretation of the longitudinal signal changes in this animal. Future studies will incorporate prospective inflammatory models with independent pathological confirmation.

Histological validation in this study was limited to qualitative hematoxylin and eosin assessment at the terminal time point. Quantitative histomorphometric analyses, such as mean linear intercept measurements, were not performed. Furthermore, precise spatial registration between histological sections and specific CT slices was not attempted due to the inherent difficulties of matching two-dimensional tissue sections to three-dimensional imaging volumes. These factors limit the strength of the imaging–histology correlation. Future studies will incorporate quantitative histopathological metrics and employ systematic co-registration strategies to enable more rigorous validation of dark-field imaging findings.

Beyond monitoring *in vivo* disease progression in mice, longitudinal dark-field CT may also support therapeutic response assessment. By leveraging within-subject measurements, it could reduce inter-animal variability that often confounds cross-sectional or pseudo-longitudinal designs. Future studies could extend this approach to additional disease models and integrate complementary modalities such as lung function testing.

Building on this preclinical foundation, extending longitudinal dark-field CT to clinical follow-up studies could further establish its value for disease surveillance and therapeutic monitoring.

In conclusion, we established a dedicated grating-based dark-field CT system and a comprehensive data processing workflow for mouse lung imaging, implementing the first laboratory-based longitudinal dark-field CT study in mice. We characterized the spatiotemporal trends of dark-field (µd) and attenuation (µ) signals across distinct induction models and observed divergent disease courses among mice subjected to the same protocol due to intrinsic inter-subject variability. The true longitudinal framework mitigates the inter-animal variability that confounds pseudo-longitudinal studies, allowing imaging signals to more faithfully reflect disease evolution and providing a noninvasive readout of pathological progression in the murine lung. Future efforts will focus on engineering a mouse-optimized small-animal CT prototype to enable large-cohort studies and statistically robust conclusions.

## Supplementary information

**Additional File 1: Fig. A1** A dedicated X-ray dark-field CT system for longitudinal mouse study. (a) Photo of the experimental setup. (b) Customized fixation device. (c) Reconstructed dark-field CT slices of a mouse lung in coronal, sagittal, and axial planes. **Fig. A2** (a) Relationship between V2N and X-ray tube voltage (kVp). (b) Relationship between the standard deviation of the dark-field CT reconstructions and the effective dose. **Fig. A3** Reconstructed slices of the attenuation and dark-field signals when the system autocorrelation length is excessively large. **Fig. A4** Attenuation and dark-field CT reconstructions of a mouse lung phantom acquired at two autocorrelation lengths. (a) excessively large autocorrelation length (3 μm); (b) appropriate autocorrelation length (0.9 μm). **Fig. C5** Left: representative reliable dark-field projection with a clean background. Right: unreliable dark-field projection exhibiting stripe artifacts (red arrows). **Fig. D6** Left: attenuation image from a commercial micro-CT; middle: attenuation from the proposed system; right: corresponding dark-field image. **Fig. D7** Left: left-lung mask; middle: right-lung mask; right: whole-lung mask.

## Data Availability

All imaging datasets, manual lung segmentation masks, and trained nnU-Net segmentation weights are available at https://github.com/XMCI/longitudinal-in-vivo-dfct-lung upon acceptance of the manuscript. In the interim, all data are available from the corresponding author on reasonable request.

## Abbreviations

µ: Linear attenuation coefficient
µ_d_: Dark-field linear diffusion coefficient
COPD: Chronic obstructive pulmonary disease
CT: Computed tomography
PBS: Phosphate-buffered saline
PMMA: Polymethyl methacrylate

## Code availability

The analysis code is available at https://github.com/XMCI/longitudinal-in-vivo-dfct-lung upon acceptance of the manuscript. In the interim, the code is available from the corresponding author on reasonable request.
