# Supplementary material for: Feasibility of longitudinal in vivo monitoring of pulmonary disease progression in mouse models using laboratory-based x-ray dark-field CT
Source: Eur Radiol Exp. 2026 Sep 8;10:120. doi: 10.1186/s41747-026-00789-w (PMC13554028; doi:10.1186/s41747-026-00789-w)
Supplement: Supplementary file 1 [file 41747_2026_789_MOESM1_ESM.docx]

# Supplementary material

# Section A. Dedicated dark-field CT system for mouse lung imaging

Building upon our existing X-ray grating–based multi-characteristic imaging system, we developed a dedicated workflow for longitudinal dark-field CT of mouse lungs, enabling high-quality image acquisition within a practical 1-hour scan time.

**[Fig. A1]** A dedicated X-ray dark-field CT system for longitudinal mouse study. (a) Photo of the experimental setup. (b) Customized fixation device. (c) Reconstructed dark-field CT slices of a mouse lung in coronal, sagittal, and axial planes.

In grating-based multi-characteristic X-ray imaging, the quality of the dark-field signal depends on the system visibility, photon counts, and the system autocorrelation length, as described in Eq. (A1)[S1]. where k represents the wavenumber of the X-ray beam. Therefore, we characterized the dependence of V^2^N on tube voltage (kVp) and its maximum tube current (mA) permitted by the system. As shown in Fig. A2, the $\text{V}^{\text{2}}\text{N}$ increases with tube voltage before peaking and subsequently decreasing. To avoid excessively high tube voltages—associated with higher radiation dose and greater device stress—we selected an operating point of 53 kVp and 500 µA for subsequent experiments.

**[Eq. A1]**	$\begin{matrix} \text{S}\text{=}\text{π}\sqrt{\text{2}\text{N}}\frac{\text{d}}{\text{p}_{\text{2}}}\text{V} \\ \text{ =}\text{π}\sqrt{\text{2}\text{N}}\frac{\text{ξ}_{\text{corr}}}{\text{λ}}\text{V} \\ \text{ =}\frac{\text{1}}{\text{2}}\text{k}\sqrt{\text{2}\text{N}}\text{ξ}_{\text{corr}}\text{V} \\ \text{ } \end{matrix}$

**[Fig. A2]** (a) Relationship between $\text{V}^{\text{2}}\text{N}$ and X-ray tube voltage (kVp). (b) Relationship between the standard deviation of the dark-field CT reconstructions and the effective dose.

In preliminary experiments, we found that selecting an appropriate system autocorrelation length is critical for obtaining high-quality dark-field images. When the autocorrelation length is too large, undesired artifacts emerge, as illustrated in Fig. A2a. The red box highlights the cardiac region, which is a solid organ without microstructural scattering, and therefore no dark-field signal should be present. However, under excessively large autocorrelation lengths, pronounced dark-field signals erroneously appear in this region, and the lung boundaries, indicated by the yellow arrow, exhibit exaggerated edge signals. This artifact arises from saturation of the dark-field signal, analogous to metal artifacts in conventional attenuation imaging. Conversely, when the autocorrelation length is too small, the dark-field signal becomes weak and less sensitive to structural variations, which is also unfavorable for longitudinal dark-field CT studies of mouse lungs. Therefore, it is essential to select an appropriate autocorrelation length that avoids signal saturation while maintaining the highest possible sensitivity to microstructural changes.

To determine an appropriate autocorrelation length, we designed a mouse-like phantom based on the anatomical dimensions of the mouse lung. The phantom body was constructed from PMMA to mimic soft tissue, while the lung region was filled with 20 µm microspheres (EPRUI, Shanghai, China) to simulate the dark-field signal of healthy mouse lungs.

**[Fig. A3]** Reconstructed slices of the attenuation and dark-field signals when the system autocorrelation length is excessively large.

We imaged the mouse phantom at different autocorrelation lengths and developed an algorithm capable of determining dark-field signal saturation directly from the projection images, thereby avoiding invalid CT acquisitions and improving experimental efficiency. Fig. A4 shows CT reconstructions of the phantom at two different autocorrelation lengths. As seen in Fig. A3, the previously described heart artifacts and artificially enhanced edge signals of the lung are no longer present at (b). At the same time, the imaging field of view was approximately 3 cm × 4 cm, sufficient to fully encompass the mouse lungs.

**[Fig. A4]** Attenuation and dark-field CT reconstructions of a mouse lung phantom acquired at two autocorrelation lengths. (a) excessively large autocorrelation length (3 µm); (b) appropriate autocorrelation length (0.9 µm).

Given the nature of longitudinal studies, the same mouse is repeatedly irradiated, making dose control essential. After determining the appropriate autocorrelation length, we further investigated the relationship between radiation dose and dark-field image quality, aiming to identify an optimal dose that ensures sufficient image quality without imposing excessive exposure. As shown in Fig. A2b, the variance of the dark-field signal decreases almost linearly with increasing effective dose. Considering both image quality and acquisition time, we selected 450 mGy as the effective dose per scan for longitudinal studies. Including additional contributions from detector readout and mechanical overhead, the total scan time was approximately one hour, resulting in an extra 90 mGy and a total dose of 540 mGy per imaging session.

# Section B. Additional methodological details

This section provides supplementary methodological details that were condensed in the main text for brevity.

**B.1 Acquisition parameters**

For each projection angle, three phase-stepped images were acquired at distinct G2 grating positions with an exposure time of 5 s per step. Dose-rate measurements were performed using a RaySafe Xi dosimeter with the R/F probe (Unfors RaySafe, Sweden), yielding 150 µGy/s. Accordingly, the total effective dose per scan was approximately 540 mGy. Under our longitudinal imaging schedule, this dose remains within acceptable limits and is not expected to exert any measurable impact on disease progression or lung histopathology.

**B.2 Pseudo-dark-field correction**

Pseudo–dark-field signals are caused by the X-ray beam hardening effect, leading to dark-field signals that are unrelated to real microstructural scattering. To estimate and correct for this effect, polymethyl methacrylate (PMMA) was selected as the calibration material. Solid PMMA is a homogeneous, amorphous polymer that does not produce small-angle scattering. Therefore, any measured dark-field signal from PMMA is attributed to spectral effects alone and treated as a pseudo-dark-field signal. This reference signal was then used to perform pixel-wise correction, thereby mitigating artifact-induced bias in the reconstructed dark-field images and enabling more accurate quantitative interpretation.

**B.3 Motion compensation**

Despite the use of custom-designed fixation tubes to immobilize the mice, gradual downward displacement of the lungs remained inevitable during prolonged upright imaging, primarily due to muscle relaxation under gravity. To compensate for this motion, we applied Otsu thresholding on the pseudo-dark-field-corrected images to segment the lung region. For a selected projection angle, the number of downward-shifted pixels was quantified and used to fit a displacement model across all projection views. In cases where Otsu thresholding failed to reliably segment the lung region, the displacement at this projection angle was manually determined and incorporated into the model. This displacement model was subsequently applied to correct lung motion on a view-by-view basis, thereby improving spatial alignment and reconstruction quality.

# Section C. Data Quality Control

We inspected dark-field projection images for every mouse at each time point. Reliable data were characterized by a clean, artifact-free background (Fig. C5 left); if all or the majority of views in a set met this criterion, the set was deemed reliable. Conversely, when most views exhibited pronounced striping artifacts (Fig. C5 right), the set was considered unreliable and excluded from subsequent quantitative analyses.

**[Fig. C5]** Left: representative reliable dark-field projection with a clean background. Right: unreliable dark-field projection exhibiting stripe artifacts (red arrows).

Stripe artifacts observed in the projection views can be attributed to multiple sources. First, system instabilities—including focal-spot jitter of the X-ray source, thermally induced expansion of the G0 grating, vibrations introduced during background acquisition, and instability of the three-step phase-stepping procedure—can perturb the fringe pattern. Second, because the laboratory setup must be frequently disassembled and reassembled, identical mechanical and thermal conditions cannot be consistently m

# Section D. Lung segmentation for mouse dark-field CT

As shown in Fig. D6, the attenuation images produced by our system exhibit markedly different texture from commercial micro-CT. Consequently, conventional mouse-lung segmentation networks trained on commercial micro-CT do not transfer and cannot be directly applied to our attenuation and dark-field images. Therefore, a segmentation model was trained on our in-house dataset.

**[Fig. D6]** Left: attenuation image from a commercial micro-CT; middle: attenuation from the proposed system; right: corresponding dark-field image.

Because the bone–lung interface is more distinct in attenuation images, we generated training labels by manually segmenting the lungs on the attenuation channel. Annotation followed a two-stage protocol: (i) coarse segmentation—for each axial slice, lung parenchyma was thresholded in ImageJ based on its low attenuation; to suppress non-pulmonary low-attenuation regions, a rough thoracic ROI was manually drawn and intersected with the thresholded map to yield a coarse mask; and (ii) contour refinement—to remove thresholding-induced holes, small non-lung fragments, and jagged boundaries, contours were extracted from the binarized coarse mask using OpenCV, size-filtered by an area threshold, and the top-ranked contours across slices were retained and redrawn. Finally, Gaussian smoothing followed by re-binarization was applied to regularize edges.

**[Fig. D7]** Left: left-lung mask; middle: right-lung mask; right: whole-lung mask.

As illustrated in Fig. D7, we first segmented the left and right lungs independently and then merged them to generate a whole-lung mask. We manually annotated 3,000 slices and fine-tuned the nnU-Net model fit our image; the trained model was subsequently applied to segment the remaining scans, yielding the longitudinal dataset used for downstream analysis. Segmentation quality was ensured through a two-stage approach. First, approximately 3,000 slices were manually annotated and used to fine-tune a pre-trained nnU-Net model [S2]. The fine-tuned model was then applied to segment all remaining scans. In the second stage, every automated segmentation underwent systematic slice-by-slice visual inspection, with manual corrections applied to approximately 10% of slices where necessary to ensure anatomical accuracy. Because all final segmentations were visually verified and corrected by an experienced operator, the quantitative results reflect human-validated masks rather than purely automated outputs. All raw data, manual lung masks, and trained model weights will be available at our GitHub repository (<https://github.com/XMCI>) upon publication.

# Supplementary References

[S1]Weber T, Bartl P, Bayer F et al: Noise in x-ray grating-based phase-contrast imaging. Med Phys 38:4133–4140 (2011). <https://doi.org/10.1118/1.3592935>

[S2]Isensee F, Jaeger PF, Kohl SAA et al: nnU-Net: a self-configuring method for deep learning-based biomedical image segmentation. Nat Methods 18:203–211 (2021). <https://doi.org/10.1038/s41592-020-01008-z>
